# Measuring the impact of multiple discrimination on depression in Europe

**DOI:** 10.1186/s12889-019-6714-4

**Published:** 2019-04-25

**Authors:** Javier Alvarez-Galvez, Antonio Rojas-Garcia

**Affiliations:** 1 Department of Biomedicine, Biotechnology and Public Health, University of Cadiz, Avda. Ana de Viya, 52, 11009 Cádiz, Spain; 2 NIHR CLAHRC North Thames, Department of Applied Health Research, University College London, 1-19 Torrington Place, London, London WC1E 7HB UK

**Keywords:** Multiple discrimination, Depression, Socioeconomic status, Multilevel analysis, Europe

## Abstract

**Background:**

The study of the health effects of perceived discrimination based on ethnic and social traits has a long-standing and widespread tradition in epidemiological research, but less attention has been paid to the study of multiple discrimination, particularly its effects on mental health. The present work aims to analyse the association between multiple discrimination and depressive symptoms in Europe, and the impact of contextual socioeconomic circumstances on this relationship.

**Methods:**

In this study, data from the 7th Round of the European Social Survey was used. Given that the outcome variable, CES-D8, is a depression scale from 0 to 24 possible values and the hierarchical organisation of individuals (level-1 units) clustered within countries (level-2 units), a linear multilevel model was carried out.

**Results:**

Our findings suggest that multiple discrimination increases our risk of suffering depressive disorder, but in addition this work provides an important step forward to explain and understand how the relationship between multiple discrimination and depression might vary depending the socioeconomic context. In particular, we can observe that differences in the prevalence of depressive symptoms along multiple discrimination levels decrease as GDP per capita increases among European countries.

**Conclusion:**

This study is relevant since provides new evidence on how the association between multiple discrimination and depression operates at the micro and macro-level context, which is fundamental to understand how macro-economic fluctuations of countries may determine depressive disorders through the effect of single and combined forms of discrimination.

## Background

Depression is one of the most common mental disorders and has been identified as a leading cause of disability [1, 2]. A recently published report from the World Health Organisation (WHO) in 2017 [3] informs that the global population suffering depression in 2015 is estimated to be 4.4%. Similarly, it has been estimated that the prevalence of depressive disorders in Europe range from 4 to 6% [3]. However, epidemiological studies have found several sociodemographic factors associated with depression, such as gender, age, marital and socioeconomic status that make certain populations more vulnerable to depressive disorders [4, 5].

Social circumstances define the context where individuals live, and thus their health and quality of life [6]. Among other social determinants such as social class, educational attainment, income distribution or material circumstances [7], studies show that discrimination has important consequences on health [8–18]. Literature shows that individuals who perceive themselves to be subjects of discrimination have a higher predisposition to suffer health problems, particularly those related to mental illness such as stress, anxiety, phobias, depression, and even high-risk health behaviours (i.e. smoking tobacco, drinking alcohol, eating disorders, suicide, etc.) that are associated to poor health outcomes [18, 19]. Perceived discrimination has also been found to be connected to specific physical health problems including hypertension, obesity, breast cancer, and other potentially risky behaviour such as substance abuse [17].

Previous research on the effects of discrimination on health have highlighted the influence of ethnic and racial discrimination on mental and physical health outcomes [19–23]. Studies has demonstrated that discriminated ethnic minorities, including those with Black, Latin, and Asian origin, are associated with larger socioeconomic inequalities and poor health outcomes [12, 24]. However, perceived discrimination based on gender, sexual orientation, language, religion, nationality, social class or disabilities can also lead to poor health outcomes [9, 25–29].

The study of the effects on health of perceived discrimination based on ethnic and social traits has a long-standing and widespread tradition in epidemiological research, but less attention has been paid to the study of multiple discrimination [30]. The concept ‘multiple discrimination’ describes the specific situation when an individual belongs to more than one disadvantaged group and therefore experiences forms of discrimination of a more complex and severe nature than those subject to discrimination on a single ground [31]. Although literature has broadly assessed the effect of single causes of discrimination, few studies have described whether multiple discrimination increases risk for poor health outcomes. A recent study using survey data from a sample of adults living in Miami (Florida), has revealed that those reporting multiple reasons for discrimination are at increased risk for major depression and subsequent depressive symptoms [32].

Two studies investigating depression across more than 20 European countries, showed that the prevalence of depressive disorders among immigrant and ethnic minorities groups was higher than among natives, which it seemed to be associated with perceived discrimination due to socio-economic conditions and ethnic and racial factors [33, 34]. These findings align with the formation of negative attitudes towards discriminated groups (e.g. immigrants), which has been found related to socioeconomic problems within countries [35]. GDP per capita, inequality, and unemployment are described in literature as contextual determinants that partially explain the variations in diverse types of social rejection. According to this perspective, discrimination might be considered as the product of competition for scarce socio-economic resources (e.g. jobs, income inequality, and social benefits). Therefore, it is essential to analyse how the association between multiple discrimination and mental health might change depending on these contextual variations.

In order to address this gap in the literature, this study aims to investigate the association between multiple discrimination and depression in Europe. Specifically, the effect of countries’ socioeconomic circumstances over the relationship between multiple discrimination and depression will be analysed. The objectives of this study are based on the following hypotheses:

- H1: The effect of multiple discrimination on depression is higher than the effect of single causes of discrimination.
- H2: The association between multiple discrimination and depression varies among European countries.
- H3. The effect of multiple discrimination on depression is lower in economically prosperous countries (i.e. those characterised by high GPD, low risk of poverty, and/or low unemployment).

## Methods

### Data and variables

With the aim of studying the association between multiple discrimination and depression in Europe, and the impact of socioeconomic context on this relationship, data from the 7th Round of the European Social Survey [36] were used. The ESS7 dataset is composed by a sample of 40,185 individuals, which are aggregated around a total of 21 countries. These countries are the following: Austria (AU), Belgium (BE), the Czech Republic (CZ), Denmark (DK), Estonia (EE), Finland (FI), France (FR), Germany (DE), Hungary (HU), Ireland (IE), Israel (IL), Lithuania (LT), the Netherlands (NL), Norway (NO), Poland (PL), Portugal (PT), Slovenia (SI), Spain (ES), Sweden (SE), Switzerland (CH), and the United Kingdom (UK). The target population of ESS7 included individuals over 15 years of age who are residents in Europe within private households, regardless of nationality, citizenship, language or legal status. For additional information, a complete description of the survey specifications can be found in the ESS7 Documentation Report [37]. In this study focused in European countries, Israel was excluded from the final sample due to comparative purposes, since we considered that socio-cultural differences might affect to the interpretation of results.

The dependent variable to be explained in this study is depression. This outcome variable has been developed through the eight-item short version of the Center for Epidemiologic Studies Depression Scale (CES-D8) [38]. The resulting indicator measures depressive symptoms defined by the American Psychiatric Association’s Diagnostic and Statistical Manual (DSM-5) for a major depressive episode in a scale whose scores range from 0 to 24, with higher values indicating a greater number of depressive symptoms [30, 31]. The item responses were assessed on a 4-point scale where 0 represents the category “rarely or none of the time”, 1 “some of the time”, 2 “most of the time”, and finally 3 “most or all of the time”. In the ESS7 questionnaire, the items who composed the scale were the following: “Using this card, please tell me how much of the time during the past week...”, (E20) “...you felt depressed?”, (E21) “...you felt that everything you did was an effort?”, (E22) “...your sleep was restless?”, (E23) “...you were happy?”, (E24) “...you felt lonely?”, (E25) “...you enjoyed life?”, (E26) “...you felt sad?”, and (E27) “...you could not get going?”. Therefore, the depression variable (CES-D8) was used as a continuous scale in which higher scores depicted a greater level or intensity of depressive symptoms [39]. The internal consistency reliability coefficients of the 8-item scale were satisfactory (Cronbach *α* = 0.79).

In the multilevel model, two types of explanatory variables were included in the analysis to adjust the relationship between multiple discrimination and depression: individual attributes and aggregate variables. At the individual level, *multiple discrimination* was the main predictor to be assessed, where 0 ‘No discrimination’, 1 ‘One reason for discrimination’, 2 ‘two or more reason for discrimination’. This variable was developed using the question of the ESS7 questionnaire ‘On what grounds is your group discriminated against?’ with the following categories of perceived discrimination based on: colour or race, nationality, religion, language, ethnic group, age, gender, sexuality, disability, or other. Following the criteria of Gayman and Barragan [32], the aggregation in three categories (i.e. no discrimination, one reason for discrimination, and two or more reasons) was aimed to increase the statistical power in the sample of people reporting discrimination.

The relationship between the depression score and multiple discrimination was adjusted by: *gender* where 0 ‘male’, 1 ‘female’; *age group* (1 ‘18–29’, 2 ‘30–39’, 3 ‘40–49’, 4 ‘50–59’, 5 ‘60–69’, 6 ‘> 70’); *education* measured by the International Standard Classification of Education (ISCED) levels: primary education or less (1 ‘ISCED 0–1’), lower secondary (2 ‘ISCED 2’), upper secondary (3 ‘ISCED 3’), post-secondary (4 ‘ISCED 4’), and tertiary education (5 ‘ISCED 5–6’); and *household total net income* in euros per month (measured in quartiles). The sample characteristics for the variables in the model are described in Table 1.

**Table 1:** Sample characteristics (*n* = 40,185)

CES-D8	Depressive symptoms	14.8% (5785)
Multiple discrimination	No discrimination	92.0% (36,961)
	One reason	5.6% (2236)
	Two or more reasons	2.5% (987)
Gender	Male	47.0% (18,870)
	Female	53.0% (21,292)
Age group	18–29 years	18.3% (7334)
	30–39 years	15.4% (6158)
	40–49 years	16.4% (6554)
	50–59 years	17.2% (6894)
	60–69 years	16.7% (6690)
	> 70 years	16.1% (6454)
Education (ISCED)	Primary education or less	10.2% (4085)
	Lower secondary	16.9% (6759)
	Upper secondary	35.8% (14,307)
	Post-secondary	14.2% (5671)
	Tertiary education	22.8% (9096)
Household income	Quartile 1	31.0% (9894)
	Quartile 2	21.9% (6983)
	Quartile 3	30.6% (9749)
	Quartile 4	16.5% (5262)

At the country level, the effect of three predictors were assessed in the multilevel models: (1) risk of poverty rate, this variable describes the share of people below 60% of the median equivalized disposable income in the country after social transfers; (2) unemployment rate, which represents the number of people unemployed as a percentage of the active population (excluding economically inactive individuals such as preschool and school children, students and pensioners); and (3) gross domestic product (GDP) per capita, as a measure of the overall size of economy for every country. These country-level data were extracted from Eurostat, the statistical office of the European Union (more information on the Eurostat database: https://ec.europa.eu/eurostat).

### Statistical analysis

Given that the dependent variable, CES-D8, is a scale from 0 to 24 possible values and the hierarchical structure of individuals (level-1 units) clustered within countries (level-2 units), a multilevel model was carried out to work with this nested structure (ICC = 0.05). Considering that we have a clustered-data structure of individuals within countries, it is statistically necessary to organize all individual units in a multilevel model of independent clusters or groups of observations [40]. This analytical solution enables the simultaneous study of variations in depression levels (CES-D8) related to individuals within their particular countries. The multilevel model can be described as follows:

$$ {Y}_{ij}={\gamma}_{00}+{\gamma}_{10}{X}_{ij}+{\gamma}_{01}{Z}_j+{\gamma}_{11}\ {Z}_j{X}_{ij}+{u}_{0j}+{e}_{ij} $$

Where the u-term *u*_*0j*_ in Eq. 1 is (random) residual error at the country level, and the e-term *e*_*ij*_ at the individual level. These errors are assumed to have mean of zero, and to be independent from the residual error. While the predictors *X*_*i*_ and *Z*_*j*_ correspond to the level 1 and level 2 respectively. In order to understand the aggregated effect of clustered-data, different models are performed. Initially, the effect of multiple discrimination is assumed to be fixed across country units, but there having a random intercept that considers the variations in responses between the countries under analysis. In a second step, the effect of multiple discrimination is assumed to vary between countries so that we can observe possible variations in the relationship between multiple discrimination and the depression score among country level units. Random intercept and random slopes model are tested in separated models to study this association. Errors are considered to be constant and are not correlated between level 2 units. Model are estimated using Restricted Maximum Likelihood.

Stata® 14.0 was used to perform data analysis, including descriptive and inferential statistics.

## Results

In order to gain a better understanding of the relationships to be modelled, Table 2 describes the mean of the depressive symptoms by countries for the five individual-level predictors. To facilitate the interpretation of this scale, the table uses a colour coding based on the highest, median, and lowest value of the distribution of each predictor across the European countries in the sample.

**Table 2:**
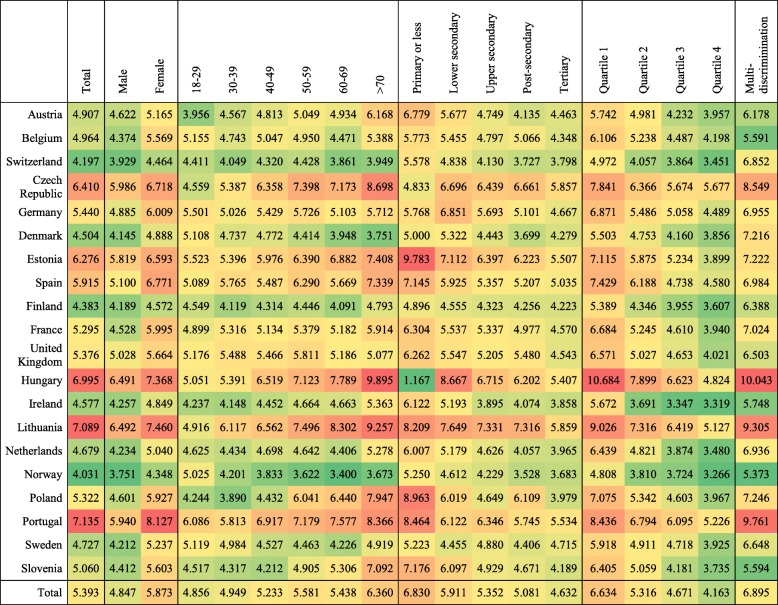
Mean depressive symptoms by countries, socio-demographic characteristics and multiple discrimination (n = 40,185)

Note: Green colour indicates a lower mean of depressive symptoms and red colour a higher mean of depressive symptoms

On average, Northern European countries such as Denmark, Finland, Norway and Sweden present lower depressive symptoms, while Southern and Eastern countries present higher scores. In particular, Portugal, Lithuania, and Hungary are the countries that show poorer results in the depression scale. With the exception of Denmark, Switzerland, and Norway, depressive symptoms seem to be more prevalent among females, older adults (in particular older groups over 70 years old), and people with low socioeconomic status (i.e. low educational attainment and low-income groups). Finally, in the last column of the table, we can observe that those who feel themselves as being discriminated by more than two grounds (i.e. people suffering multiple discrimination) present a higher mean score in the CES-D8 scale. On average, multiple discrimination increases mean depressive symptoms in a 28% among European countries, but this percentage can be even doubled depending on the country (e.g. in Hungary, the mean score of depressive symptoms is 6.995, but this score increases to 10.043 for those with multiple discrimination).

To answer our initial research hypothesis, Table 3 presents the results of six random intercept multilevel models with main effects and cross-level interactions between level-1 and level-2 predictors. Models 1 to 3 includes the individual-level predictors (i.e. multiple discrimination, gender, age, education, and income), and step by step the effect of separated level 2 predictors on the depression score are added. Cross-level interactions are included in models 4 to 6. At the individual level, multiple discrimination (i.e. being discriminated for two or more reasons) shows a positive correlation with the depression scale (CES-D8), which means that combined types of discrimination may determine higher levels of depressive symptoms. The association between multiple discrimination and the depression score is statistically significant across all the models (with coefficients between 2.055 and 3.392), even when controlling by socio-demographics and socioeconomic predictors. As previously observed, depressive symptoms increase among females (0.807–0.808), when respondents age increases (in particular in the 50–59 age group, 0.622), and for those with lower socioeconomic status, i.e. in low education level (− 0.340) and low-income groups (− 1.147).

**Table 3:** Results for random intercept multilevel models

Variable	Model 1	Model 2	Model 3	Model 4	Model 5	Model 6
One reason discrimination	1.629^***^ (0.099)	1.629^***^ (0.099)	1.629^***^ (0.099)	1.166^***^ (0.350)	1.497^***^ (0.222)	1.994^***^ (0.425)
Two or more reasons	2.055^***^ (0.166)	2.056^***^ (0.166)	2.057^***^ (0.166)	2.547^***^ (0.579)	2.504^***^ (0.399)	3.392^***^ (0.702)
Female	0.807^***^ (0.044)	0.807^***^ (0.044)	0.806^***^ (0.044)	0.807^***^ (0.044)	0.807^***^ (0.044)	0.808 (0.044)
30–39	0.192^***^ (0.079)	0.193^***^ (0.079)	0.192^***^ (0.079)	0.192^***^ (0.079)	0.193^***^ (0.079)	0.191^***^ (0.079)
40–49	0.463^***^ (0.077)	0.463^***^ (0.077)	0.463^***^ (0.077)	0.4634^***^ (0.077)	0.464^***^ (0.077)	0.461^***^ (0.077)
50–59	0.620^***^ (0.075)	0.620^***^ (0.075)	0.620^***^ (0.075)	0.620^***^ (0.075)	0.621^***^ (0.075)	0.617^***^ (0.075)
60–69	0.210^***^ (0.076)	0.211^***^ (0.076)	0.210^***^ (0.076)	0.209^***^ (0.076)	0.212^***^ (0.076)	0.205^***^ (0.076)
> 70	0.546^***^ (0.081)	0.547^***^ (0.081)	0.547^***^ (0.081)	0.544^***^ (0.081)	0.547^***^ (0.081)	0.542^***^ (0.081)
Lower secondary	−0.336^***^ (0.093)	− 0.335^***^ (0.093)	− 0.336^***^ (0.092)	− 0.338^***^ (0.092)	− 0.335^***^ (0.092)	− 0.339^***^ (0.092)
Upper secondary	−0.926^***^ (0.088)	− 0.925^***^ (0.088)	− 0.926^***^ (0.088)	− 0.927^***^ (0.088)	−0.925^***^ (0.088)	− 0.927^***^ (0.088)
Post-secondary	−0.981^***^ (0.098)	−0.980^***^ (0.098)	− 0.979^***^ (0.098)	−0.983^***^ (0.098)	− 0.979^***^ (0.098)	−0.982^***^ (0.098)
Tertiary	−1.114^***^ (0.094)	−1.113^***^ (0.094)	−1.113^***^ (0.094)	−1.116^***^ (0.094)	−1.113^***^ (0.094)	−1.114^***^ (0.094)
Quartile 2	−1.149^***^ (0.062)	− 1.150^***^ (0.062)	− 1.150^***^ (0.062)	− 1.150^***^ (0.062)	− 1.150^***^ (0.062)	− 1.151^***^ (0.062)
Quartile 3	−1.620^***^ (0.059)	−1.621^***^ (0.059)	− 1.621^***^ (0.059)	−1.621^***^ (0.059)	− 1.621^***^ (0.059)	−1.623^***^ (0.059)
Quartile 4	−2.102^***^ (0.073)	−2.102^***^ (0.073)	−2.102^***^ (0.073)	−2.103^***^ (0.073)	−2.102^***^ (0.073)	−2.103^***^ (0.073)
Risk of poverty	0.088^***^ (0.026)			0.088^***^ (0.027)		
One reason * Risk Pov.				0.021 (0.015)		
Two or more * Risk Pov.				−0.022 (0.025)		
Unemploy.		0.066 (0.039)			0.066 (0.039)	
One reason * Unemploy.					0.014 (0.021)	
Two or more * Unemploy.					−0.050 (0.040)	
GDP per capita			−0.023^***^ (0.004)			−0.023^***^ (0.004)
One reason * GDP						−0.003 (0.004)
Two or more reasons * GDP						−0.012^**^ (0.006)
Constant	4.412^***^ (0.610)	5.722^***^ (0.434)	8.890^***^ (0.493)	4.429^***^ (0.612)	5.721^***^ (0.434)	8.890^***^ (0.492)
Level 2 (sig_u)	−0.317^**^ (0.165)	−0.152 (0.164)	−0.565^***^ (0.167)	−0.316^*^ (0.165)	− 0.152 (0.164)	−0.565^***^ (0.167)
Level 1 (sig_e)	1.315^***^ (0.004)	1.315^***^ (0.004)	1.315^***^ (0.004)	1.315^***^ (0.004)	1.315^***^ (0.004)	1.315^***^ (0.004)
Log likelihood	−8.0e+ 04	−8.0e+ 04	−8.0e+ 04	−8.0e+ 04	−8.0e+ 04	−8.0e+ 04
Wald Chi Square	2985.123	2975.934	3005.669	2988.161	2978.154	3010.905
AIC	1.6e+ 05	1.6e+ 05	1.6e+ 05	1.6e+ 05	1.6e+ 05	1.6e+ 05
BIC	1.6e+ 05	1.6e+ 05	1.6e+ 05	1.6e+ 05	1.6e+ 05	1.6e+ 05
Countries	20	20	20	20	20	20

Note: Significance level ^***^ *p* < 0.01, ^**^ *p* < 0.05, ^*^ *p* < 0.10. Standard errors in parenthesis (n = 40,185)

In order to study if the association of multiple discrimination and the prevalence of depressive symptoms is weaker in economically prosperous countries (H3), three level 2 predictors were included at the aggregated level: risk of poverty (model 1), unemployment (model 2), and GDP per capita (model 3) over individual-level depressive symptoms. As we can observe, risk of poverty at the macro-level is positively associated (0.088) to the prevalence of depressive symptoms (model 1), and, on the contrary, aggregated GDP per capita is negatively related (− 0.023) to depressive symptoms (model 3). Macro-level unemployment was not found associated to depressive symptoms in model 2 (0.066). These results indicate that economic prosperity –defined by either a low risk of poverty or higher GDP per capita – is positive for mental health but contextual unemployment is rather neutral. Therefore, favourable socioeconomic circumstances at the country-level might decrease the rate of depressive symptoms among European population.

With the aim to find out whether the relationship of multiple discrimination on depressive symptoms varies along contextual socioeconomic conditions, models 4–6 include the interaction terms between the level 2 predictors and multiple discrimination at the individual-level. Cross-level interactions were only statistically significant in model 6 where the effect of multiple discrimination and GDP per capita were combined (− 0.012; *p* < .05), but interactions in models 4–5 were not. Consistent with hypotheses H2 and H3, GDP moderates the effect of multiple discrimination on the prevalence of depressive symptoms, which means that mental inequalities based related to multiple discrimination will be lower in wealthier countries.

Table 4 shows similar results for random slope coefficient models, although a higher variability in the model decrease the level of significance in some models (e.g. model 4 and 5). However, in model 6 we can observe the same pattern to that found in Table 3, i.e. the differences in CES-D8 scale between multiple discrimination levels decrease as GDP per capita increases.

**Table 4:** Results for the random slope coefficient models

Variable	Model 1	Model 2	Model 3	Model 4	Model 5	Model 6
One reason disc.	1.606^***^ (0.383)	1.603^***^ (0.430)	1.629^***^ (0.303)	1.297 (1.362)	1.600^*^ (0.905)	1.715^**^ (1.090)
Two or more reasons	2.077^***^ (0.414)	2.098^***^ (0.458)	2.137^***^ (0.339)	2.579^*^ (1.450)	2.451^**^ (0.970)	4.188^***^ (1.257)
Female	0.808^***^ (0.044)	0.808^***^ (0.044)	0.808^***^ (0.044)	0.808^***^ (0.044)	0.808^***^ (0.044)	0.808^***^ (0.044)
30–39	0.193^***^ (0.079)	0.193^***^ (0.079)	0.193^***^ (0.079)	0.193^***^ (0.079)	0.193^***^ (0.079)	0.192^***^ (0.079)
40–49	0.462^***^ (0.077)	0.463^***^ (0.077)	0.462^***^ (0.077)	0.462^***^ (0.077)	0.463^***^ (0.077)	0.461^***^ (0.077)
50–59	0.622^***^ (0.075)	0.623^***^ (0.075)	0.622^***^ (0.075)	0.622^***^ (0.075)	0.623^***^ (0.075)	0.621^***^ (0.075)
60–69	0.206^***^ (0.076)	0.207^***^ (0.076)	0.207^***^ (0.076)	0.207^***^ (0.076)	0.208^***^ (0.076)	0.206^***^ (0.076)
> 70	0.545^***^ (0.081)	0.546^***^ (0.081)	0.545^***^ (0.081)	0.545^***^ (0.081)	0.546^***^ (0.081)	0.544^***^ (0.081)
Lower secondary	−0.340^***^ (0.093)	−0.340^***^ (0.093)	− 0.339^***^ (0.092)	− 0.340^***^ (0.093)	−0.340^***^ (0.093)	− 0.339^***^ (0.092)
Upper secondary	−0.924^***^ (0.088)	−0.925^***^ (0.088)	− 0.924^***^ (0.088)	−0.924^***^ (0.088)	− 0.924^***^ (0.088)	−0.923^***^ (0.088)
Post-secondary	−0.981^***^ (0.098)	−0.980^***^ (0.098)	− 0.978^***^ (0.098)	−0.980^***^ (0.098)	− 0.979^***^ (0.098)	− 0.978^***^ (0.098)
Tertiary	−1.112^***^ (0.094)	−1.112^***^ (0.094)	−1.110^***^ (0.094)	−1.112^***^ (0.094)	−1.112^***^ (0.094)	−1.110^***^ (0.094)
Quartile 2	−1.147^***^ (0.062)	− 1.147^***^ (0.062)	− 1.148^***^ (0.062)	− 1.147^***^ (0.062)	−1.147^***^ (0.062)	− 1.148^***^ (0.062)
Quartile 3	−1.618^***^ (0.059)	−1.618^***^ (0.059)	− 1.619^***^ (0.059)	−1.618^***^ (0.059)	− 1.618^***^ (0.059)	− 1.620^***^ (0.059)
Quartile 4	−2.095^***^ (0.073)	−2.095^***^ (0.073)	−2.096^***^ (0.073)	−2.095^***^ (0.073)	−2.094^***^ (0.073)	−2.096^***^ (0.073)
Risk of poverty	0.086^***^ (0.026)			0.088^***^ (0.041)		
One reason * Risk Pov.				0.014 (0.060)		
Two or more * Risk Pov.				−0.023 (0.063)		
Unemploy.		0.056 (0.037)			0.066 (0.058)	
One reason * Unemploy.					0.001 (0.085)	
Two or more * Unemploy.					−0.38 (0.092)	
GDP per capita			−0.027^***^ (0.004)			−0.023^***^ (0.006)
One reason * GDP						−0.001 (0.009)
Two or more reasons * GDP						−0.018^**^ (0.011)
Constant	4.457^***^ (0.623)	5.809^***^ (0.463)	9.323^***^ (0.523)	4.424^***^ (0.931)	5.716^***^ (0.629)	8.855^***^ (0.708)
Level 2 (sig_u)	0.120^**^ (0.140)	0.244^*^ (0.128)	−0.143^***^ (0.156)	−0.118^*^ (0.139)	−0.240^*^ (0.128)	−0.185^***^ (0.157)
Level 1 (sig_e)	1.314^***^ (0.004)	1.314^***^ (0.004)	1.314^***^ (0.004)	1.314^***^ (0.004)	1.314^***^ (0.004)	1.314^***^ (0.004)
Log likelihood	−8.0e+ 04	−8.0e+ 04	−8.0e+ 04	−8.0e+ 04	−8.0e+ 04	−8.0e+ 04
Wald Chi Square	2520.983	2504.387	2571.585	2521.525	2504.820	2581.643
AIC	1.6e+ 05	1.6e+ 05	1.6e+ 05	1.6e+ 05	1.6e+ 05	1.6e+ 05
BIC	1.6e+ 05	1.6e+ 05	1.6e+ 05	1.6e+ 05	1.6e+ 05	1.6e+ 05
Countries	20	20	20	20	20	20

Note: Significance level ^***^ p < 0.01, ^**^ p < 0.05, ^*^ p < 0.10. Standard errors in parenthesis (n = 40,185)

**Fig. 1:**
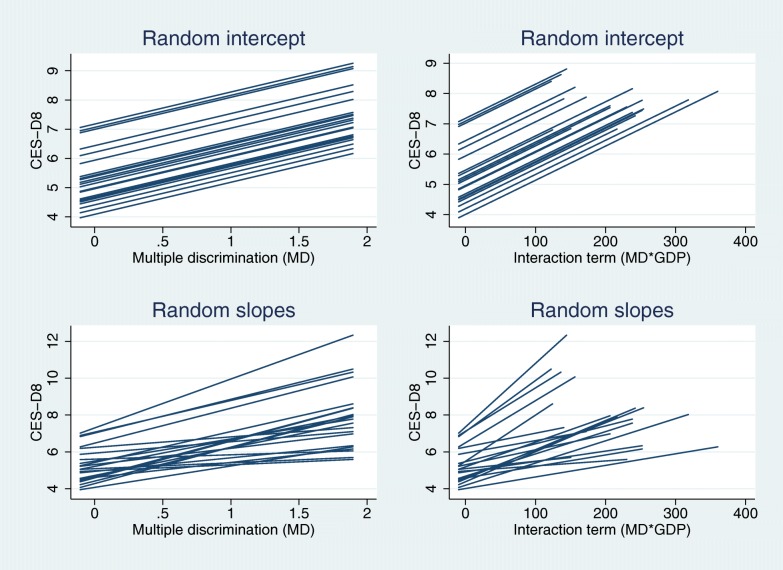
Association between depressive symptoms and multiple discrimination in random intercept models and random slope models for main effect and crosslevel interactions

To have a better understanding of the moderation effect of GDP per capita on the association of multiple discrimination on depressive symptoms, this relationship has been described in Fig. 1. Random intercepts models for single and interaction effects are depicted above, and random slope models are described below. These graphs confirm that the effect of multiple discrimination on depressive symptoms is higher than the effect of single causes of discrimination (H1), the association between multiple discrimination and the depression scale significantly differs among European countries (H2), and the impact of multiple discrimination on depressive symptoms is lower in countries having favourable socioeconomic circumstances, in particular GPD per capita (H3).

**Fig. 2:**
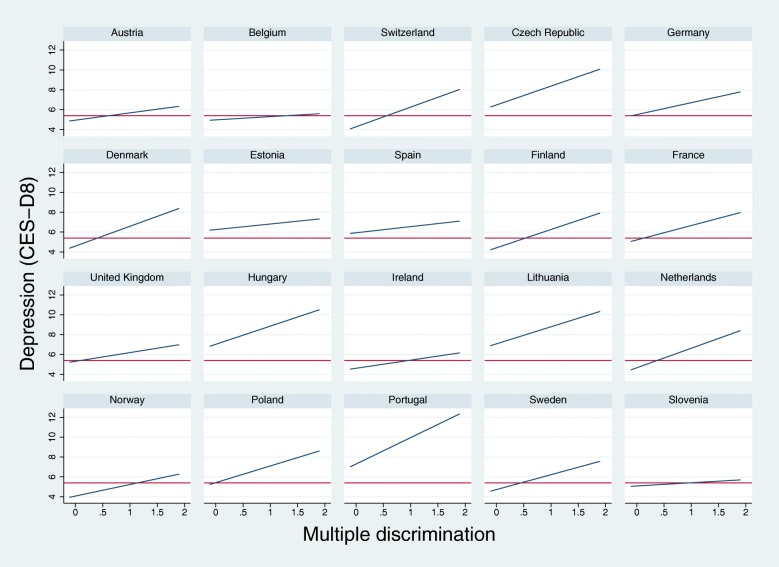
Random slope model for the relationship between multiple discrimination and depression by European countries. Note: red lines identify the average depressive symptoms for every country; blue lines defines the direction of the effects

Finally, for a better identification of the relationship between multiple discrimination and the prevalence of depressive symptoms among European countries, the main effect has been plotted in Fig. 2. According to the initial descriptive results in Table 2, the stronger effect of multiple discrimination on the depression scale has been found in the East and South European countries, and this relationship is particularly strong in countries such as Hungary, Czech Republic, Lithuania and Portugal.

## Discussion

Although there is a vast amount of literature on discrimination and mental health, some of the main issues on this topic remain unclear. This study aimed to investigate three questions around the relationship between multiple discrimination and depression: i) how is multiple discrimination associated with depression in Europe, using a continuous measure of depressive symptoms (CES-D8); ii) how this relationship varies among European countries; and specifically, iii) how the moderation effect of countries’ socioeconomic circumstances influence the relationship between multiple discrimination and depressive symptoms. To answer these research questions a multilevel strategy has been implemented to explain and understand the negative relationship of multiple discrimination on depression in Europe according contextual socioeconomic differences. Overall, findings show that countries from Northern Europe present lower rates of depressive symptoms; multiple discrimination is positively associated to depressive symptoms, and macro-level variables such as GDP, risk of poverty and unemployment have correlated with depression scores in different ways.

As hypothesised, our findings suggest that multiple discrimination may increase the risk of suffering depressive disorder, these results support what has been found in previous research [41, 42]. In addition, this study show that the relationship between multiple discrimination and depression may be context-dependent. In this regard, the association may vary depending on the socioeconomic context. In particular, differences in the prevalence of depressive symptoms across multiple discrimination levels decrease as GDP per capita increases. However macro-level unemployment was not statistically significant in any cross-level interaction between multiple discrimination and risk of poverty, which is possibly due to the lower variability in these macro-indicators that might affect to polarised groups (i.e. basically unemployed and those in risk of poverty). Although further evidence is needed to answer this analytical inconsistency, one possible explanation is that macro-level variations in unemployment and risk of poverty rates do not affect the whole population but, in particular, those groups at the bottom of social hierarchy. In any case, our results would be consistent with what has been investigated in other studies [32, 41–43].

Aligned with previous research, the present study has explored how multiple discrimination may be a risk factor for depression [32], however, it also provides an important step forward to explain and understand how the relationship between multiple discrimination and depressive symptoms might vary in relation to other variables. For instance, the effect of multiple discrimination seems to be lower in wealthier European countries [31]. These findings have important implications to understand how the negative effect of combined forms of discrimination and subsequent stigmatisation might affect in countries characterised by socioeconomic problems (e.g. Eastern and Southern ones), and specifically in those that have been severely affected by the recent economic crisis [20].

Despite factors related to depression have been largely studied, the impact of multiple discrimination seems relatively unexplored and it has only been in recent years when it has drawn the attention of researchers [32]. The impact of multiple discrimination on depression should be breaking down in future research in order to assess the partial influence that may have on depressive disorders. Apart from socioeconomic, and racial and ethnic discrimination, our findings point out that other added forms of discrimination may have a negative impact on the people’s mental health across Europe, especially in those countries less economically developed.

The notion of stigma has been defined as ‘the phenomenon whereby an individual with an attribute is deeply discredited by his/her society and is rejected as a result of the attribute […] a process by which the reaction of others spoils normal identity’ [44]. This definition consider stigma as a consequence of a single socially rejected characteristic, but then what about those individuals that are socially labelled by multiple discrediting characteristics? Although there seem to be contextual variations in the association between multiple discrimination and the prevalence of depressive symptoms, this study shows that being discriminated by multiple reasons increases the risk of depressive symptoms in all European countries. Therefore, the harmful effects of multiple discrimination on psychological wellbeing and mental health should be considered to understand the subsequent impact on the quality of life of stigmatised groups [45]. All in all, we must consider that some individuals with discredited attributes can also have high self-esteem and good mental health, they can be happy and resilient to social discrimination experiences [46]. However, according our findings on the positive relationship between multiple discrimination and depressive symptoms, we must consider that resilience against discrimination feelings might be slowly undermined if the load of discrediting labels grows.

This study presents two main limitations that should be addressed in future research. On the one hand, the linear causation between multiple discrimination and depressive symptoms cannot be established since this work is based in cross-sectional data. For instance, reverse causation could also be possible (i.e. mentally ill people might present a higher predisposition to perceive discrimination). Studies has generally focused on perceived discrimination as a social determinant of health outcomes, but as recent works have demonstrated this theoretical assumption might be inverted as well [47]. In addition, although in this study we have considered a single-general measure of multiple discrimination, future works should address how do different types of discrimination are integrated in a single individual profile and how do different combinations of perceive discrimination might increase or reduce the risk of mental illness [41]. Evidence show that people suffering discrimination present different and specific problems. People with disabilities, LGBTI, immigrants, lower classes or the elderly, are different social groups characterized by specific norms, values, and social interest, thus future research should identify the concerns of concrete groups in context but also considering that multiple discredited characteristics can determine the health and wellbeing of single individuals.

## Conclusions

The present study shows that the positive association between multiple discrimination and the prevalence of depressive symptoms in Europe might vary depending on the economic prosperity of every country. This finding provides new evidence on how this complex association operates at the micro and macro-level context, which is fundamental to understand how macro-economic fluctuations of countries may determine depressive disorders through the effect of single and combined forms of discrimination. Having this mechanism in mind, the future orientation of EU health and social policies should contribute to reduce the impact of social and economic inequalities in mental health in the most vulnerable groups, and especially to protect discriminated social groups against the lagging effects of the recent financial crisis.
